# Immune Checkpoint Inhibitor-Associated Myocarditis: A Literature Review

**DOI:** 10.7759/cureus.52952

**Published:** 2024-01-25

**Authors:** Rohail Gul, Muhammad Shehryar, Anber Mahboob, Hira K Kareem, Arslan Inayat, Danish Safi, Amir Kamran

**Affiliations:** 1 Internal Medicine, Shifa Tameer-E-Millat University Shifa College of Medicine, Islamabad, PAK; 2 Internal Medicine, King Edward Medical University, Lahore, PAK; 3 Internal Medicine, Sharif Medical and Dental College, Lahore, PAK; 4 Internal Medicine, HSHS St. Marys Hospital, Decatur, USA; 5 Hematology and Medical Oncology, West Virginia University School of Medicine, Morgantown, USA; 6 Hematology and Medical Oncology, Charleston Area Medical Center, Charleston, USA

**Keywords:** review, multiple myeloma treatment, immune therapy mediated myocarditis, immune-checkpoint inhibitor adverse effects, checkpoint inhibitor therapy

## Abstract

Recently in the field of oncology, immune checkpoint inhibitors (ICI) are being increasingly utilized both in clinical trials and in clinical practice. It is a form of biological therapy that targets tumors by activating the immune system, which in turn eliminates proliferating cancer cells. These have numerous immune-related adverse events (irAEs), one of which is myocarditis, which has high rates of mortality. This article was a narrative review of myocarditis related to ICI use.

Studies from the PubMed, Cochrane, and American Society of Clinical Oncology (ASCO) databases were used in writing this review. The databases were searched for original publications for adverse effects related to ICI use and myocarditis specifically. There are numerous published instances of cancer immunotherapy causing myocarditis. ICI therapy has numerous benefits, as it upregulates the immune system to target cancer cells, utilizing the body's own defense mechanisms to target proliferating cells. Myocarditis is a serious side effect, however. Therefore, on balance, these monotherapies are worth using. While this literature review primarily identifies cross-reaction as the main mechanism of myocarditis, there are other possible mechanisms. One proposed mechanism involves a shared antigen between the myocardial tissue and the tumor. This mechanism is called molecular mimicry, where the monoclonal antibody attacks both the myocardial tissue and the tumor cell.

Management of ICI-induced myocarditis has not been studied by randomized controlled trials or prospective studies, but based on previous case reports and case series it is mostly treated with steroids initially. An ICI rechallenge after temporary discontinuation appears conceivable in many cases, especially given its therapeutic effects, but only limited data are available on the safety of a rechallenge after an irAE. The lack of RCTs regarding rechallenge with an ICI after irAE, more so specifically about myocarditis, along with the overall results and the complexity involved in such cases once again emphasize the need to make decisions on an individual basis by a multidisciplinary expert working group. At the same time, the focus should also be on publishing more data as the need will grow along with the indications for ICI therapies.

## Introduction and background

Immune checkpoints are a normal part of the immune system. Their role is to prevent an immune response from being so strong that it destroys healthy cells in the body. Immune checkpoints engage when proteins on the surface of immune cells called T cells recognize and bind to partner proteins on other cells, such as some tumor cells. These proteins are called immune checkpoint proteins. When the checkpoint and partner proteins bind together, they send an “off” signal to the T cells. This can prevent the immune system from destroying the cancer. Immunotherapy drugs called immune checkpoint inhibitors work by blocking checkpoint proteins from binding with their partner proteins [1]. This prevents the “off” signal from being sent, allowing the T cells to kill cancer cells. One such drug acts against a checkpoint protein called CTLA-4. Other immune checkpoint inhibitors act against a checkpoint protein called PD-1 or its partner protein PD-L1. Some tumors turn down the T cell response by producing lots of PD-L1.

Myocarditis associated with immune checkpoint inhibitors (ICIs) has a reported incidence of 0.04% to 1.14%. However, in comparison to other immune-related adverse events (irAEs), it exhibits a significantly higher mortality rate ranging from 25% to 50%. Moreover, the use of combination ICI therapy shows nearly twice the incidence and mortality in myocarditis, even though it remains a relatively uncommon adverse event compared to other irAEs.

Myocarditis is a serious condition where inflammation develops in the myocardium of the heart wall. Symptoms include fatigue, fever, shortness of breath, chest pain, rapid or irregular heartbeat, and light-headedness. The causes of acute myocarditis are broadly classified into infectious and non-infectious categories. In most cases (50%), no cause can be identified (idiopathic). Infectious causes include viruses (coxsackie, HIV, adenovirus), bacteria (staphylococci, salmonella, shigella, legionella), parasites (trichinosis, schistosomiasis), protozoa (Trypanosoma cruzi, Toxoplasma gondi), spirochetes (Borrelia burgdorferi). Non-infectious causes include eosinophilic myocarditis, systemic lupus erythematosus, polymyositis, dermatomyositis, sarcoidosis, inflammatory bowel disease, acute rheumatic fever, venoms and medications which in our case, immune checkpoint inhibitors [2].

The management of ICI-induced myocarditis lacks examination through randomized controlled trials or prospective studies. Treatment relies on insights from past case reports and case series, primarily involving initial steroid administration. The type and dosage depend on the severity of the myocarditis. Patients are also treated with conventional cardiac therapy in these cases.

The primary goal of this paper is to provide a comprehensive literature review on ICI-associated myocarditis. The authors aim to summarize existing knowledge on the incidence, mechanisms, and management of myocarditis as an immune-related adverse event (irAE) associated with the use of ICIs in cancer treatment. The paper aims to discuss the incidence of myocarditis related to various types of ICIs, such as antibodies targeting programmed cell death protein-1 (PD-1), programmed cell death Ligand (PD-L1), and cytotoxic T-lymphocyte-associated protein 4 (CTLA-4).

The focus is on understanding the mechanisms, including cross-reaction and molecular mimicry, which lead to myocarditis. The paper includes specific cases and examples of patients who developed myocarditis during or after ICI therapy. These cases provide real-world scenarios, showcasing the diversity of cancers and ICI treatments associated with myocarditis. We discuss the management of ICI-induced myocarditis, emphasizing the lack of randomized controlled trials (RCTs) and the reliance on case reports and series for current management strategies. The paper outlines the importance of early administration of high-dose corticosteroids and the necessity to discontinue ICIs based on the severity of myocarditis.

We also want to emphasize the need for more research and data publication related to ICI-induced myocarditis. They suggest that as the indications for ICI therapies grow, more knowledge of mechanisms and management is essential.

## Review

Immunotherapies

There are several types of cancer immunotherapies that target specific antibodies: There are antibodies targeting programmed cell death protein-1 (PD-1), programmed cell death ligand (PD-L1), and cytotoxic T-lymphocyte associated protein 4 (CTLA-4) checkpoints. Medications targeting PD-1 receptors include pembrolizumab, nivolumab, cemiplimab, atezolizumab, durvalumab, and avelumab. There are numerous reports in the literature that describe severe myocarditis following ICI use, with the highest frequency amongst PD-1 and PDL-1/CTLA-4 therapies. Ipilimumab, nivolumab, and pembrolizumab have been associated with higher rates of myocarditis [1].

Ipilimumab is a monoclonal antibody that is FDA-approved for melanoma and is undergoing clinical trials for non-small cell carcinoma, small cell lung cancer, bladder cancer, and metastatic prostate cancer [3]. CTLA-4 is a protein receptor that downregulates the immune system. Ipilimumab binds to CTLA-4, blocking its action on immune cells, therefore, allowing cancer cells to replicate unchecked. Ipilimumab binds to CTLA-4, blocking its action on immune cells, therefore, allowing immune cells to target proliferating cancer cells. Ipilimumab leads to myocarditis by cross-reacting with myocardial tissue [4]. Other mechanisms are described below. 

Nivolumab is a monoclonal antibody used to treat melanoma, lung cancer, malignant pleural mesothelioma, renal cell carcinoma, and Hodgkin lymphoma amongst others. The final effect of this medication is that it allows T cells to kill cancer cells through a series of steps. T cells have a receptor called PD-1 on its surface. If PDL-1 binds to PD-1, the T-cell becomes active. This is a mechanism meant to suppress the immune system to avoid overreaction. Cancer cells produce PD-L1 which ultimately inactivates T cells by preventing an attack on tumor cells. Nivolumab blocks PD-L1, therefore, allowing T cells to function and kill cancer cells. Similar to ipilimumab, nivolumab cross-reacts with myocardial tissue, resulting in myocarditis [3]. 

Pembrolizumab is a humanized antibody that treats melanoma, lung cancer, head and neck cancer, Hodgkin lymphoma, gastric carcinoma, cervical cancer, and breast cancer. Pembrolizumab has a similar mechanism to nivolumab and blocks PD-1, allowing T cells to kill cancer cells. Its effect on myocardial tissue is similar to the previously two mentioned monoclonal antibodies [5].

Mechanisms

While this literature review primarily identifies cross-reaction as the main mechanism of myocarditis, there are other possible mechanisms. One proposed mechanism involves a shared antigen between the myocardial tissue and the tumor. This mechanism is called molecular mimicry, where the monoclonal antibody attacks both the myocardial tissue and the tumor cell. One animal study with cynomolgus monkeys indicates that utilizing certain monoclonal antibodies leads to upregulation of CD4+ and CD8+ T-cells, which in turn infiltrates the heart [6].

ICI therapy has numerous benefits, as it upregulates the immune system to target cancer cells, utilizing the body's own defense mechanisms to target proliferating cells. Myocarditis is a serious side effect, however, these monotherapies are useful in the context of cancer treatment. Common side effects of myocarditis include decreased ejection fraction, chest pain, fatigue, swelling of the legs, rapid or irregular heartbeat, and shortness of breath. If these side effects are severe, the immunotherapy infusions can be discontinued and corticosteroids can be given to treat the myocarditis, as exemplified in the case report above. Alternatively, more traditional forms of cancer therapy such as chemotherapy can be used. Chemotherapy has its own side effects, so the decision to discontinue newer therapies is not easy to make. In summary, numerous cancer immunotherapies can cause myocarditis, a side effect that clinicians and patients should consider when deciding to initiate the infusions [7].

There are numerous published articles on cancer immunotherapy causing myocarditis. In one instance, Afzal et al. treated a 72-year-old man with metastatic BRAF melanoma with ipilimumab (3mg/kg, 4 infusions) and nivolumab (1 mg/kg, four infusions followed by 3 mg/kg every two weeks) [8]. Three infusions resulted in several side effects including dyspnea, peripheral edema, and a weight gain of 10 kg. Echo showed reduced ejection fraction (EF) from 50% to 15%. A cardiac biopsy revealed interstitial inflammation with lymphocytes and interstitial fibrosis, a hallmark of myocarditis. Corticosteroids were initiated at 1 mg/kg orally and the patient's EF ultimately increased to 40% after two months [8].

Adverse events

ICIs have been reported to cause myocarditis in a variety of cancers. One such case was reported after a 70-year-old Japanese female with advanced hepatocellular cancer was initiated on atezolizumab plus bevacizumab therapy [8]. Another case was reported when a 71-year-old Asian man with metastatic renal cell cancer on ipilimumab and nivolumab combination therapy presented with symptoms of rapidly worsening cardiogenic shock [9]. Similarly, it has also been reported in a 51-year-old woman with triple-negative breast cancer with a history of modified radical mastectomy after she received chemotherapy combined with PD-1 inhibitors [10]. Myocarditis was diagnosed in a patient with chordoma, who was treated with third-line sintilimab combined with anlotinib, and in a 69-year-old female with advanced non-small cell lung cancer, who was treated with combined bevacizumab and camrelizumab [11]. Another case of myocarditis was reported in choroidal malignant melanoma with liver and bone metastases, which was treated with an anti-PD-1 antibody nivolumab [12], durvalumab, and tremelimumab were prescribed in an advanced endometrial cancer case, which resulted in myocarditis [13]. Active myocarditis in a 69-year-old man with primary liver cancer and a 75-year-old man with non-small-cell lung cancer after treatment with camrelizumab has also been reported [14]. Sintilimab-induced myocarditis has been reported in non-small cell lung cancer [15] while nivolumab-induced myocarditis has been reported in squamous cell cancer of the lung [16]. According to one case report, pembrolizumab caused myocarditis when used in the treatment of nasopharyngeal cancer [17]. Fulminant myocarditis has also occurred in a case of relapsed chronic myelomonocytic leukemia after the use of ipilimumab [18]. Nivolumab has also been associated with myocarditis when used to treat thymoma [19]. Also, a case of autoimmune myocarditis during pembrolizumab treatment for uveal metastatic melanoma has been reported [20].

**Figure 1:**
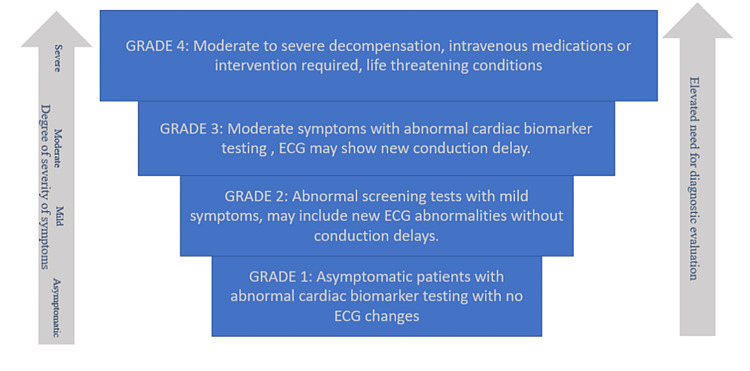
Grading of myocarditis due to immune checkpoint inhibitors This has been adapted from the American Society of Clinical Oncology clinical practice guidelines [22]. The guideline was written in collaboration with the National Comprehensive Cancer Network (NCCN) for the management of immune-related adverse events (irAEs). The figure shows the increasing severity of symptoms of myocarditis with the use of immune checkpoint inhibitors for the treatment of various malignancies.

Immune checkpoint inhibitors, including anti-CTLA-4 or anti-PD-1/ PD-1 L, have a very good response against various malignancies. Besides treating the malignancy, they have been associated with irAEs. The combination of ipilimumab and nivolumab is frequently used in the treatment of melanoma. In comparison to nivolumab monotherapy, this combination therapy has been related to fatal myocarditis more often [21] as listed in Figure 1 along with other case reports and series that document ICI myocarditis (Table 1).

**Table 1:** Cases with immune checkpoint inhibitor-induced myocarditis

Author	Year	Title	Cancer type	Age (in years) and sex of patients	Immunotherapy used
Iwasaki et al. [5]	2021	A case of immune checkpoint inhibitor-associated myocarditis after initiation of atezolizumab plus bevacizumab therapy for advanced hepatocellular carcinoma.	Advanced hepatocellular carcinoma	75 F	Atezolizumab plus bevacizumab
Miyauchi et al. [9]	2021	Myocarditis as an immune-related adverse event following treatment with ipilimumab and nivolumab combination therapy for metastatic renal cell carcinoma: a case report.	Metastatic renal cell carcinoma	71 M	Ipilimumab and nivolumab
Yang et al. [10]	2022	Severe immune-related hepatitis and myocarditis caused by PD-1 inhibitors in the treatment of triple-negative breast cancer: a case report.	Triple-negative breast cancer	51 F	PD-1 inhibitors
Chen et al. [11]	2021	Myocarditis related to immune checkpoint inhibitors treatment: two case reports and literature review.	(i) chordoma; (ii) non-small cell lung carcinoma	(i) 71 M; (2) 69 F	(i) Sintilimab combined with anlotinib; (ii) pemetrexed combined with bevacizumab and camrelizumab
Tadokoro et al. [12]	2016	Acute Lymphocytic Myocarditis With Anti-PD-1 Antibody Nivolumab.	Choroidal malignant melanoma	69 F	Nivolumab
Mahmood et al. [13]	2018	Myocarditis with tremelimumab plus durvalumab combination therapy for endometrial cancer: A case report	Endometrial carcinoma	75 F	Tremelimumab plus durvalumab
Zhang et al. [14]	2022	Immune-related myocarditis in two patients receiving camrelizumab therapy and document analysis.	(i) primary liver cancer; (ii) non-small cell lung carcinoma	(i) 69 M; (ii) 75 M	Camrelizumab
Bi et al. [15]	2021	Immune checkpoint inhibitor-induced myocarditis in lung cancer patients: a case report of sintilimab-induced myocarditis and a review of the literature.	Non-small cell lung carcinoma	68 M	Sintilimab
Semper et al. [16]	2016	Drug-induced myocarditis after nivolumab treatment in a patient with PDL1- negative squamous cell carcinoma of the lung.	Squamous cell carcinoma of the lung	75 M	Nivolumab
Wang and Hu [17]	2019	Successful therapy for autoimmune myocarditis with pembrolizumab treatment for nasopharyngeal carcinoma.	Nasopharyngeal carcinoma	45 M	Pembrolizumab
Berg et al. [18]	2017	Immune-related fulminant myocarditis in a patient receiving ipilimumab therapy for relapsed chronic myelomonocytic leukemia.	Relapsed chronic myelomonocytic leukemia	66 M	Ipilimumab
Chen et al. [19]	2018	Fatal myocarditis and rhabdomyolysis induced by nivolumab during the treatment of type B3 thymoma.	Thymoma	43 M	Nivolumab
Läubli et al. [20]	2015	Acute heart failure due to autoimmune myocarditis under pembrolizumab treatment for metastatic melanoma.	Uveal metastatic melanoma	73 F	Pembrolizumab

irAEs have been categorized into four different grades with every next grade depicting the augmenting severity of adverse events (Figure 1). Myocarditis, like other irAEs, has also been classified into four grades depending on the severity of symptoms and diagnostic laboratory markers. Grade 1 includes asymptomatic patients with no ECG abnormalities, however, only the cardiac biomarker testing is abnormal. Grade 2 myocarditis is associated with milder symptoms alongside abnormal cardiac biomarker testing. It may include new ECG abnormalities without any conduction delay. Grade 3 is linked with abnormal cardiac biomarker testing with moderate symptoms or the ECG may reflect changes with new conduction delay. Grade 4 is the most life-threatening of all the grades with moderate to severe decompensation for which immediate intervention is needed [22, 23]. Symptoms for myocarditis include chest pain, dyspnea, palpitations, and fatigue. Signs may include pleural effusion, arrhythmias, peripheral pitting edema, S3 gallop, and jugular venous distension. It can also transform into fulminant myocarditis with impaired ventricular function, hypotension, and cardiogenic shock [24].

The management of ICI-induced myocarditis has not been studied by randomized controlled trials or prospective studies, however, based on previously published case reports and case series, it is mostly treated with steroids initially. According to the American Society of Clinical Oncology (ASCO), ICI should be held immediately for grade-1 myocarditis and permanently discontinued in grade 2-4 myocarditis [22]. ICI-induced myocarditis is divided into four grades as per ASCO guidelines with grade 1 being the mildest (asymptomatic biomarker elevation or imaging abnormalities) and grade 4 being the most severe (Table 2). Initial management should be early administration of high-dose corticosteroids (1-2 mg/kg of oral or IV prednisolone) and in the absence of immediate improvement with this regimen, add 1 g of IV methylprednisolone daily along with other immunosuppressive agents (mycophenolate, infliximab, or anti-thymocyte globulin) [22]. Some cardiologists have also suggested to treat it with aggressive initial steroid therapy with IV methylprednisolone 500-1000 mg daily until clinical stability followed by oral prednisolone at 1 mg/kg daily tapered over four to six weeks [25, 26]. Patients should also be treated with conventional cardiac therapy in these cases [23].

**Table 2:** Immune checkpoint inhibitor-induced myocarditis per ASCO guidelines Adapted from Gürdoğan et al. [4]

Grade	Clinical Findings	Intervention
Grade 1	· Abnormal ECG findings OR · Increased cardiac biomarkers	· Discontinue ICI · Cardiac monitoring + Follow-up · Treat symptoms, if present
Grade 2	Mild symptoms with: · Increased cardiac biomarker OR · Abnormal ECG findings OR · Abnormal ECHO findings	· Discontinue ICI permanently · Cardiac monitoring + Follow-up · In case of increasing troponin level or new changes on ECG, transfer the patient to cardiac ICU · Initiate high-dose methylprednisolone orally or intravenously (1-2 mg/kg) according to clinical symptoms and troponin levels. Continue this regimen until condition improves till Grade 1, then taper the dose within 4-6 weeks.
Grade 3	· Symptoms on mild activity OR · Moderately impaired screening tests	· Same as Grade 2
Grade 4	· Signs of decompensation	· Same as Grade 2 · If no response to high-dose steroid therapy within 24 hours, consider other immunotherapies e.g infliximab, mycophenolate, anti-thymocyte globulin, plasmapheresis · Mechanical circulatory support and/or inotropic therapy should be started

Patients on CPI therapy can develop myocarditis presented as arrhythmias, along with other systemic conditions such as myositis, myasthenia gravis, and vasculitis [25]. Patients with 10-30% involvement of body surface area need to hold CPI therapy [27].

According to current oncological guidelines, most grade 2 or higher irAE require systemic corticosteroid therapy and temporary or permanent discontinuation of ICIs. Specifically, permanent discontinuation of ICIs is recommended for the most severe irAEs (Common Terminology Criteria for Adverse Events grade 4) [22, 28, 29]. A reintroduction of ICI after a temporary hold seems plausible in multiple instances, particularly considering its therapeutic benefits. However, there is only limited available data regarding the safety of such a reintroduction after an irAE. The 2021 ASCO guideline update regarding the management of irAEs in patients treated with ICI therapy does not give any guidance about rechallenge with the ICIs [30]. Moreover, limited information is available on the safety of a rechallenge with an ICI after an irAE and even less is available specific to myocarditis.

A retrospective study in Japan focused specifically on the recurrence of irAEs after ICI resumption. In this study, 42 out of 64 patients (65.6%) resumed ICIs after discontinuation; additionally, 33 of 42 patients received the same ICIs, and all received anti-PD-1 or anti-PD-L1 agent monotherapy after irAEs. A total of 13 patients (31.0%) had any grade irAEs. Of these 13 patients with irAE recurrence, six (14.3%) developed the same irAEs as the initial ones, and seven (16.7%) experienced other irAEs. Analysis of factors revealed that age, sex, and primary tumor type/site were not associated with recurrence [31]. Another cohort study, the largest to date, using WHO’s VigiBase reported a 28.8% recurrence rate of the same irAE associated with the discontinuation of ICI therapy after a rechallenge with the same ICI. In a rechallenge, colitis, hepatitis, and pneumonitis had higher recurrence rates compared with other irAEs. Available data for myocarditis was comparable to other irAE with rechallenge in 102 (1.7%) versus 345 (1.9%) cases who did not get a rechallenge. When the rates of recurrence were stratified by the organs involved in the initial irAE myocarditis recurred in zero of three patients [32]. A randomized control trial on outcomes of ICI rechallenge in NSCLC revealed ICI rechallenge had less severe toxicity than initial ICI treatment [33].

The absence of RCTs on rechallenges with an ICI following irAEs, particularly in the context of myocarditis, underscores the importance of approaching decisions on a case-by-case basis. The overall findings and intricacies in managing such cases highlight the necessity for decisions to be made by a multidisciplinary expert working group.

## Conclusions

In conclusion, this literature review sheds light on the emerging concern of ICI-associated myocarditis, a serious adverse event with potential high mortality rates. The use of ICIs in cancer immunotherapy has demonstrated significant benefits in activating the immune system to target cancer cells. However, the review emphasizes the need for vigilance and awareness of the potential for myocarditis, among other irAEs.

The mechanisms underlying ICI-induced myocarditis, particularly cross-reaction and molecular mimicry, are discussed, highlighting the complex interactions between monoclonal antibodies and myocardial tissue. The reported cases presented in the article underscore the diverse range of cancers and ICI therapies associated with myocarditis, emphasizing the importance of monitoring and early detection.

Management strategies for ICI-induced myocarditis are outlined, emphasizing the role of corticosteroids as the initial treatment. The grading system for myocarditis severity, as per the ASCO guidelines, provides a framework for decision-making regarding ICI discontinuation and intervention intensity. The article also touches upon the potential for rechallenge with ICIs after myocarditis, acknowledging the limited data available and the need for individualized, multidisciplinary decisions.

Overall, while recognizing the therapeutic benefits of ICIs, the review underscores the importance of considering the risk of myocarditis and other irAEs in the context of cancer treatment. The multidisciplinary approach to decision-making, coupled with the publication of more data, is emphasized as crucial for managing these complex cases. As the use of ICIs continues to grow, ongoing research and collaboration among healthcare professionals will be essential to better understand, prevent, and manage ICI-associated myocarditis for the benefit of cancer patients.
